# Local protection bubbles: an interpretation of the slowdown in the spread of coronavirus in the city of São Paulo, Brazil, in July 2020

**DOI:** 10.1590/0102-311XEN109522

**Published:** 2023-12-15

**Authors:** Jose Paulo Guedes Pinto, Patrícia Camargo Magalhães, Gerusa Maria Figueiredo, Domingos Alves, Diana Maritza Segura Angel

**Affiliations:** 1 Universidade Federal do ABC, São Bernardo do Campo, Brasil.; 2 Universidad Complutense de Madrid, Madrid, España.; 3 Instituto de Medicina Tropical, Universidade de São Paulo, São Paulo, Brasil.; 4 Faculdade de Medicina de Ribeirão Preto, Universidade de São Paulo, Ribeirão Preto, Brasil.; 5 Escola de Engenharia de São Carlos, Universidade de São Paulo, São Carlos, Brasil.

**Keywords:** Social Distancing, COVID-19, Virus Shedding, Distanciamento Social, COVID-19, Eliminação de Partículas Virais, Distanciamiento Social, COVID-19, Esparcimiento de Virus

## Abstract

After four months of fighting the pandemic, the city of São Paulo, Brazil, entered a phase of relaxed social distancing measures in July 2020. Simultaneously, there was a decline in the social distancing rate and a reduction in the number of cases, fatalities, and hospital bed occupancy. To understand the pandemic dynamics in the city of São Paulo, we developed a multi-agent simulation model. Surprisingly, the counter-intuitive results of the model followed the city’s reality. We argue that this phenomenon could be attributed to local bubbles of protection that emerged in the absence of contagion networks. These bubbles reduced the transmission rate of the virus, causing short and temporary reductions in the epidemic curve - but manifested as an unstable equilibrium. Our hypothesis aligns with the virus spread dynamics observed thus far, without the need for ad hoc assumptions regarding the natural thresholds of collective immunity or the heterogeneity of the population’s transmission rate, which may lead to erroneous predictions. Our model was designed to be user-friendly and does not require any scientific or programming expertise to generate outcomes on virus transmission in a given location. Furthermore, as an input to start our simulation model, we developed the COVID-19 Protection Index as an alternative to the Human Development Index, which measures a given territory vulnerability to the coronavirus and includes characteristics of the health system and socioeconomic development, as well as the infrastructure of the city of São Paulo.

## Resumo

Após quatro meses lutando contra a pandemia, a cidade de São Paulo, Brasil, entrou em uma fase de flexibilização das medidas de distanciamento social em julho de 2020. Simultaneamente, houve queda na taxa de distanciamento social e redução no número de casos, mortes e ocupação de leitos hospitalares. Um modelo de simulação multiagente foi desenvolvido para entender a dinâmica da pandemia na cidade de São Paulo. Ao contrário do esperado, os resultados contraintuitivos do modelo acompanharam a realidade da cidade. Argumentamos que este fenômeno pode ser atribuído às bolhas locais de proteção que surgiram na ausência de redes de contágio. Estas bolhas reduziram a taxa de transmissão do vírus, causando reduções curtas e temporárias na curva epidêmica - mas se manifestaram como um equilíbrio instável. Nossa hipótese está alinhada com a dinâmica da propagação do vírus observada até o momento, sem a necessidade de suposições *ad hoc* sobre limiares de imunidade coletiva natural ou heterogeneidade da taxa de transmissão da população, o que pode levar a previsões errôneas. Nosso modelo foi projetado para ser fácil de usar e não requer nenhum conhecimento científico ou de programação para gerar resultados sobre a transmissão do vírus em um determinado local. Além disso, como insumo para iniciar nosso modelo de simulação, desenvolvemos o Índice de Proteção contra a COVID-19 como alternativa ao Índice de Desenvolvimento Humano, que mede a vulnerabilidade de um determinado território ao coronavírus e inclui características do sistema de saúde e do desenvolvimento socioeconômico, além da infraestrutura da cidade de São Paulo.

## Resumen

Tras cuatro meses luchando contra la pandemia, la ciudad de São Paulo, Brasil, empezó una fase de flexibilización de las medidas de alejamiento social en julio de 2020. A la vez, hubo una reducción en la tasa de alejamiento social y en el número de casos, muertes y ocupación de camas en los hospitales. Se desarrolló un modelo de simulación multiagente para entender la dinámica de la pandemia en la ciudad de São Paulo. Diferente de lo esperado, los resultados contradictorios del modelo reflejaron la realidad de la ciudad. Sostenemos que se puede atribuir este fenómeno a las burbujas locales de protección que surgieron durante la ausencia de redes de contagio. Estas burbujas redujeron la tasa de transmisión del virus, reduciendo de forma corta y temporal la curva epidémica -pero se manifestaron como un equilibrio inestable. Nuestra hipótesis se alinea con la dinámica de la propagación del virus observada hasta el momento, sin la necesidad de suposiciones ad hoc sobre umbrales de inmunidad colectiva natural o heterogeneidad de la tasa de transmisión de la población, lo que puede provocar previsiones equivocadas. Nuestro modelo se proyectó para ser fácil de usar y no necesita ningún conocimiento científico o de programación para generar resultados sobre la transmisión del virus en un determinado local. Además, como insumo para iniciar nuestro modelo de simulación, desarrollamos el Índice de Protección contra la COVID-19 como una alternativa al Índice de Desarrollo Humano, que mide la vulnerabilidad de un determinado territorio al coronavirus e incluye características del sistema de salud y del desarrollo socioeconómico, además de la infraestructura de la ciudad de São Paulo.

## Introduction

Since the first case was reported on February 26th in the city of São Paulo, Brazil ^1^, the spread of the pandemic has demonstrated a complex dynamic in Brazil. Despite this alarming scenario, many states, municipalities, and even neighborhoods were pressured to reopen the economy (even before the reopening of public parks) in July 2020. The state of São Paulo relaxed the social distancing measures it had implemented ^2^ on June 1st, when the pandemic was just reaching the inland cities. The outcomes of this policy were to be expected: each reopening phase was followed by an increase in the number of deaths. At the end of July, the COVID-19 epidemic in the state of São Paulo reached its most critical stage. However, there was one interesting exception: the city of São Paulo. In July, after entering a more relaxed stage of social distancing, the city’s social distancing rate fell along with the number of new cases, deaths, and hospital beds occupied ^3^. Looking closely at the data ^4^ on daily cases, deaths, and bed availability, they all decrease from the beginning of July 2020 to the beginning of October 2020. The data on cases oscillates, but when you look at the 7-day average, the downtrend is clear.

Along with these phenomena, eight seroprevalence surveys were published for the city of São Paulo, which revealed a maximum of 12% of the population immune at the end of June ^5^.

This information sparked a public debate aimed at unveiling the cause of this effect in the city ^6^, with at least three hypotheses in vogue: the positive outcome of non-pharmaceutical interventions (NPI), herd immunity, and protective bubbles accompanied by the exhaustion of the social-contagion network.

According to the first explanation, the positive effect in the city of São Paulo can be attributed to the non-pharmaceutical interventions carried out by the population, which includes social distancing, hand washing, and protective masks wearing, even though these protocols were only partially adopted and not mandatory ^7^. However, some researchers argue that this alone does not fully explain why the number of hospitalizations did not increase again in the city of São Paulo with the relaxation of social distancing measures.

The second explanation suggests that social or herd immunity can be achieved even with low immunization levels among the population. On the one hand, other authors ^8^ ^,^ ^9^ ^,^ ^10^, analyze studies that show that there may in fact be fewer people susceptible to the coronavirus due to other defenses in the body that can fight this virus. In addition to neutralizing antibodies and T-cells, CD4+ and CD8 cells have also been identified as potential defenders. There are studies ^11^ ^,^ ^12^ ^,^ ^13^ investigating heterogeneity within the population, which could lead to a decrease in the percentage of the population that must be infected to achieve herd immunity, thereby leading to a decrease in the infection rate. Using Britton et al. ^11^ parameters, the effective herd immunity threshold could be reduced to 43% (or even 34%, depending on the scenario), whereas Gomes et al. ^12^ shows that this number could drop to 20%. However, the authors of the first study explicitly highlighted that their estimates “*should be interpreted as an illustration of how population heterogeneity affects herd immunity rather than as an exact value or even a best estimate*” ^11^ (p. 846). Britton et al. ^11^ emphasized the limitations of the study.

The third explanation - our hypothesis - is developed throughout this article. In short, we do not need to assume collective immunity (or fewer people being susceptible to the coronavirus), nor ignore the fact that people are easing their social-distancing practices. Based on the multi-agent model - coronavirus dispersion model (MD Corona), we argue that this phenomenon is due to local bubbles of protection that emerge in the city of São Paulo in the absence of contagion networks. These bubbles slowdown the spread of coronavirus, causing short and temporary reductions in the epidemic curve - but they manifest as an unstable equilibrium.

Notably, a study ^14^ incorporated social interaction layers into complex networks to comprehensively capture the intricacies of the pandemic’s dynamics. Furthermore, the study made a prediction regarding a decrease in the number of infections in July 2021. However, given the intricate nature of this model, it is not feasible for an end-user without scientific and programming knowledge to manipulate it and obtain results according to their specific location.

## Methods

To simulate the SARS-CoV-2 epidemic curve, we developed a model called coronavirus dispersion model (MD Corona), based on a multi-agent model ^15^. The main objective of this simulator is to provide users with a straightforward tool for simulating the epidemic curve in neighborhoods and communities with different vulnerabilities connected to large urban centers from their own smartphones or computers (https://acaocovid19.org/simulador/territorios).

This model is inspired by the original virus model ^16^ found in the modeling environment NetLogo ^17^, which is supported by the work of Yorke et al. ^18^. Unlike susceptible-infected-recovered (SIR) or susceptible-exposed-infected-recovered (SEIR) models ^19^ ^,^ ^20^ and statistical-model approaches, the multi-agent model ^21^ ^,^ ^22^ does not rely on pandemic data (such as cases, deaths, and recoveries) to make predictions about the epidemic curve.

In our multi-agent model ^15^, a number of individuals (agents) are moving randomly (up/down/left/right) across a two-dimensional spatial grid composed of 41×41 parcels. The agents can be displaced anywhere in the parcels.

### How does the model work?

To initiate the simulation, the user must first define the population density, the percentage of social distancing, and the COVID-19 Protection Index (CPI) ^23^, by consulting a table provided in the simulator that displays information of various neighborhoods or districts in Brazilian cities.

The model’s time scale is set in days, with each round equivalent to one day. The agents, which move randomly within the environment, are categorized into one of three states: healthy agents (green), infected agents (red), or immune agents (grey). During simulation (activated by clicking on “reset” and then “start”), the transmission of the virus is determined by the probability of an encounter between at least two individuals on the grid and the probability of infection according to their states.

The number of infected individuals is depicted in a graph, along with the number of people who have become immune (immunity curve). A counter tracks the number of simulation days, as well as the percentage of infected, immune, and deceased individuals within the population. The simulation speed can be adjusted by the user using a slider.

This model provides a straightforward explanation for why the spread of the virus decreased in some cities, despite the reduction in social distancing policies. We demonstrate that this phenomenon is the result of an unstable equilibrium created by “local protection bubbles”, and the depletion of contagion networks. Complex models require high scientific knowledge, and it is difficult to assess the impact of specific variables on the dynamics of virus transmission.

### Model parameters

The dynamics of the spread of the coronavirus are determined by epidemiological constants including: (i) the period of virus transmission; (ii) the immunity period; (iii) the initial number of infected individuals; and (iv) the infection fatality rate (IFR). The variable parameters that can be configured by the user to describe different scenarios are: (v) the number of individuals in the grid; (vi) the probability of transmitting the virus between individuals; and (vii) the practice of social distancing.

Below, we will define each of these seven factors and justify the choice of values based on the medical literature available at the time of the study. As an open-source code, users can modify these values. These parameters are summarized in the Supplementary Material (https://cadernos.ensp.fiocruz.br/static//arquivo/supl-e00109522_8056.pdf).

The period of virus transmission (i) was set at 18 days due to the wide range of variation in this period, which depends on the disease severity. This was computed as the isolation period of 14 days recommended by the World Health Organization ^24^ added to the mean incubation period of the virus (4 days), since there are reports of virus transmission during this period ^25^ ^,^ ^26^ ^,^ ^27^ ^,^ ^28^.

Although the duration of effectiveness of COVID-19 vaccines against the disease decreases somewhat by 6 months after full immunization ^29^, the effects of the vaccines and the immunity time of the vaccine virus were not considered in this research.

Establishing an average (ii) immunity period for SARS-CoV-2 presents many issues. Some studies indicate that the antibody response varies depending on the length of the infection period and the severity of the disease ^30^. Therefore, they do not provide an average immunity length. Other studies ^31^ ^,^ ^32^ ^,^ ^33^ ^,^ ^34^ report that antibody responses to other human coronaviruses (SARS-CoV, MERS-CoV, alpha- and beta-coronaviruses) wane over time, varying from 12 weeks to 34 months. Based on this literature, the immunity period for an agent is assumed to be 180 days.

The number of individuals (v) in the grid (or the population density) is an important parameter in the model, since it affects the frequency of contact between agents in the grid and, consequently, the probability of the virus transmission between infected and healthy individuals. To make the simulator more user-friendly, in the version available on our website, we have converted the “number of people” variable in the grid into a “demographic density” variable (set with sliders), which enables the user to easily set the simulator to a territory of their choice. The coefficient that converts “number of people” to “demographic density” is defined using a calibration process, the methodology of which is discussed below.

The initial number of infected individuals (iii) was fixed at one person, since one agent represents 33,604 people, which is the minimum number required for the virus transmission dynamics to start, regardless of the total number of people in the grid. The model also allows for a reintroduction of a new infected agent periodically (the timing of which depends on the scenario we want to simulate). This enables the emergence of new infection waves, and the system remains open, which is consistent with the reality of the virus circulation between different territories.

In our model, the epidemic curve encompasses both symptomatic and asymptomatic patients and (vi) the probability of transmiting the virus between individuals depends on several non-pharmaceutical interventions, such as wearing masks, hygiene procedures, and social distancing measures. But it is also affected by the social condition and the particularities of the territory, such as the existence of basic sanitation, the average number of people per household, and the ability of families to implement social distancing measures. This is a mixture of territorial, health, and social factors that are not easy to quantify. We parameterize them in the simulator using an effective transmission probability denominated the CPI, which is an innovation developed by our research group ^23^ ^,^ ^35^ and is based on the Surroundings Index (SI) methodology ^36^.

The Human Development Index (HDI) is widely acknowledged, but is a poor measurement of the vulnerability of different territories to the coronavirus. In contrast, the CPI considers the characteristics of the health system, human development, and territorial indicators, which is a more appropriate index to describe the vulnerability of a given territory to coronavirus transmission when compared to HDI. Both HDI and CPI are divided into five levels: very high (0.8-1), high (0.7-0.799), medium (0.6-0.699), low (0.5-0.599), and very low (0-0.499). which measures the vulnerability of the neighborhood or city ^36^. The effective probability and the scale of HDI/CPI are defined using calibration.

Another important feature of this model is the inclusion of the social distancing rate (vii) as a dynamic parameter. By restricting the movement of some agents, this parameter impedes the virus spread, and can be modified during the simulation to reflect changes in social distancing over time.

Furthermore, the (iv) IFR determines the lethality of the disease. It is calculated as the proportion between the number of infected people and the number of deaths, including asymptomatic and undiagnosed cases. The IFR is also influenced by local health conditions, such as bed occupancy and hospital accessibility, as well as age factors. Several estimates of the COVID-19 fatality rate for Brazil have been made based on the total number of deaths and seroprevalence surveys. Mallapaty ^37^ sampled 25,025 participants from all 27 Brazil’s Federative Units ^38^ and suggested an IFR of 1%. However, a more accurate seroprevalence survey ^5^ indicated an IFR of 0.7% in the city of São Paulo. In our model, we chose this measurement as a constant for all territories, since our objective is to simulate the virus spread in urban regions.

A detailed “how to use” the simulator is available in the Supplementary Material (https://cadernos.ensp.fiocruz.br/static//arquivo/supl-e00109522_8056.pdf).

### Model calibration

To calibrate the model ^39^, it was necessary to establish a conversion coefficient between the number of agents in the grid and the population density of a given territory. To achieve this, the probability of virus transmission was modified until it aligned with the same percentage of infected individuals found in the seroprevalence survey on a certain date for the city of São Paulo. These tests detect the presence of immunoglobulin G (IgG) antibodies produced by people who have been infected with the SARS-CoV-2 for at least 20 days ^5^.

For example, in the case of the city of São Paulo, which has a population density of 8,054.7 inhabitants per km^2^, we set the number of agents in the grid at 369 and the effective transmission probability at 40% to reach 9.38% of the infected population (data from seroprevalence surveys) ^5^. The simulation also considers the known history of social distancing ^40^ over the time period specified in Table 1. As a result, the CPI scale indicates a high level (0.79), where the social distancing rate is the average of the daily isolation rates during the period indicated in the date column.

**Table 1:** Timeline of social distancing.

Period	Description	Social distancing rate (%)	Days (n)
25/Feb to 17/Mar	1st case and self-imposed social distancing	27	21
18/Mar to 21/Mar	Officially imposed social restrictions	43	4
22/Mar to 12/Apr	Decline in isolation	58	22
13/Apr to 03/May	Further decline	53	21
04/May to 31/May	Announcement of the *Plano São Paulo*	51	28
01/Jun to 22/Jun	*Plano São Paulo* - seroprevalence survey 9.5%	48	22
23/Jun to 12/Jul	Reduced non-pharmaceutical interventions phase	46	20
Total simulated days	-		138

Source: São Paulo’s State Government ^2^.

By establishing these crucial parameters, the simulation results reveal that, after calibration, approximately 9.38% of the population in São Paulo had been infected, on average, with 0.08% of the percentage of deaths (or IFR of 0.75%) recorded on June 22, 118 days after the first reported case.

These outcomes are consistent with the seroprevalence (immunity) surveys ^5^, which reported that 9.5% (with a 1.7% error interval) of São Paulo’s population had been infected by COVID-19 on the same date.

As with all multi-agent models, the MD Corona model depends on initial conditions that randomly determine the relative positions of infected and immobilized individuals. To account for this variability, we ran all the simulated scenarios 100 times using a Python program and examined the average results. A more comprehensive stochastic analysis could investigate the different trends in the simulation results and identify the positions of the behavioral change thresholds, which are indicated by inflections in the curve.

Finally, it is important to highlight that the Intelligent Monitoring and Information System of São Paulo (SIMI-SP) computes the social distancing rate by using anonymous information on displacements within the mapped municipalities in São Paulo, provided by telephone companies Vivo, Claro, Oi, and TIM via the Brazilian Association of Telecommunications Resources (ABR Telecom) and the Institute for Technological Research (IPT).

The social distance rate is based on the locations obtained by cell phone antennas (Base Transceiver Stations - BTSs). These antennas “mark” a reference for the place where the cell phone “slept” from 10:00p.m. to 2:00a.m. and identify whether during the day the cell moves from this reference. However, this measurement is sensitive to errors, since it does not consider people who work night shifts or who do not have a cell phone, or other cell phone companies.

### Calibration coefficient

**Figure 1:**
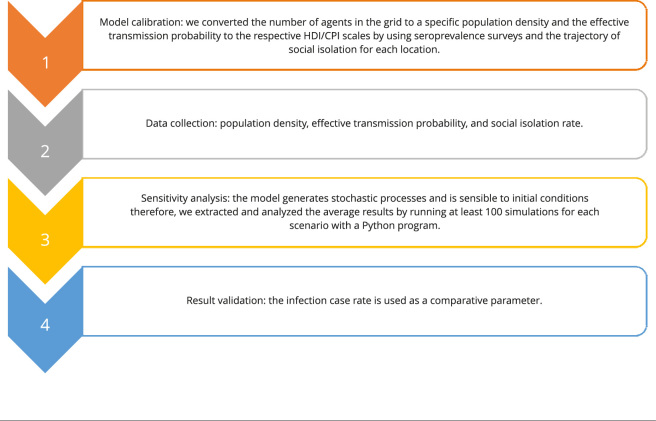
Summary of the main steps developed in this research for each simulation.

CPI: COVID-19 Protection Index; HDI: Human Development Index.

As mentioned above, the calibration coefficient converts the number of agents into demographic density. The number of people in the grid is calculated by multiplying the demographic density of a specific territory by 0.0498. Our model is calibrated to simulate a range of population densities from a minimum of 3,010 inhabitants per km^2^ to a maximum of 20,080 inhabitants per km^2^, with the number of agents in the grid ranging from 150 to 1,000. This flexible calibration allows us to adjust the MD Corona parameters to reflect the pandemic dynamics in different territories. Finally, Figure 1 summarizes the main steps developed in this research for each simulation.

## Results

Using the calibrated model, we can make predictions about the epidemic curve and explore different scenarios. The dynamics of the multi-agent simulator describe a closed system, with interactions between agents in the same environment.

### Scenario 1: reducing social distancing

To investigate the effect of reopening the economy on the evolution of the epidemic curve, we reduce the social distancing rate to 20% in Scenario 1.

**Figure 2:**
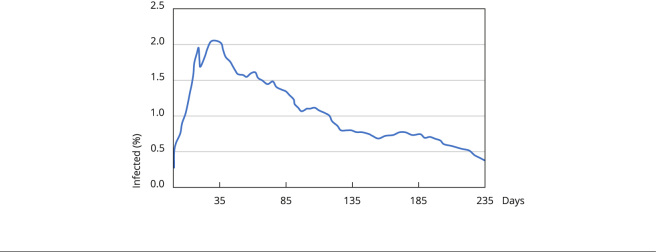
Results of the simulation using coronavirus dispersion model (MD Corona) for Scenario 1, considering the social distancing rates for the city of São Paulo, Brazil, as shown in Table 1 and extended by 100 days with a 20% social isolation rate.

We applied the official social distancing rate timeline released by the São Paulo State Government (Table 1) and added the hypothesized 20% reduction in social distancing rate for the next 100 days, starting on July 12. After 238 simulation days from the first recorded case of the virus, the average curve of infected people showed a 12.71% infection rate among the agents and a death rate of 0.12% (or an IFR of 0.9%), as shown in Figure 2.

The simulations showed a decrease in the infection curve, even with a drastic reduction in social-distancing, with a slight and temporary increase after the 150th day. This outcome seems counterintuitive, given the low immunity rate. Therefore, we developed other hypotheses to explain this effect.

**Figure 3:**
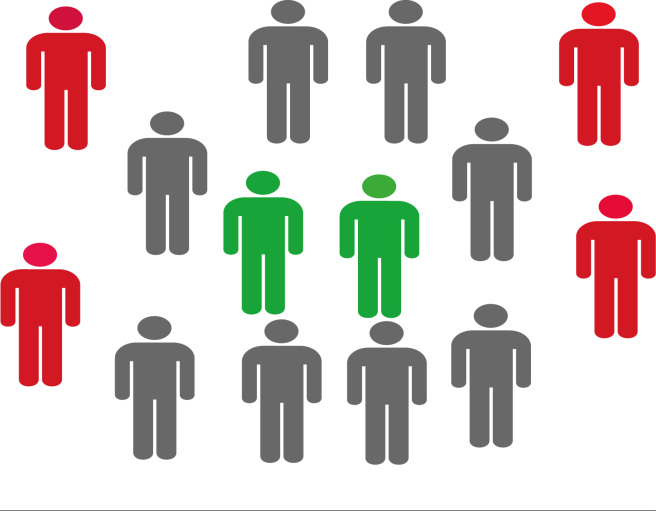
Graphical representation of a group that was exposed to the virus, which eventually exhausted the infection network, thus creating local protection.

Note: health agents (green); infected agents (red); and immune agents (gray).

Figure 3 provides a possible explanation for this effect. It shows that there are local bubbles of protection against coronavirus transmission, where infected agents (red) are surrounded by immune ones (gray) who protect susceptible agents (green) from infection. This concentration of infected agents surrounded by immune ones may be responsible for the decrease in the infection curve, despite the reduction in social distancing.

It is noteworthy that the hypotheses regarding protection bubbles are local effects. Therefore, they differ from the herd immunity hypothesis, which suggests that the virus can be suppressed throughout the entire environment, resulting in a global and stable equilibrium. Our hypotheses suggest a local equilibrium that may be unstable and could be disrupted by the introduction of a new infected agent, which would burst these bubbles and reinitiate the infection networks. However, this dynamic applies exclusively to a density compatible with the city of São Paulo.

### Scenarios 2 and 3: reintroducing one sick agent

Working with the situation as it stood on July 12, we then reintroduced one sick agent, representing 0.27% of São Paulo’s population (33,604 people) on the 138th day. We reintroduced an infected agent only on the 138th day because we started our prediction from that day. In addition, we already know the number of cases and deaths before this period. One agent was chosen because it is the minimum value that can claim the continuous infection dynamic in the model regardless of the number of agents in the grid.

The random reintroduction (within the model’s spatial environment) of one infected agent has the effect of bursting protection bubbles, resulting in another wave of transmission.

**Figure 4:**
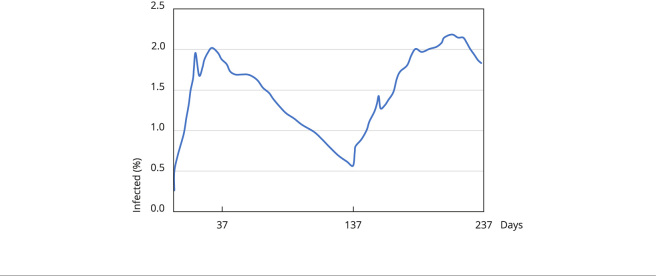
Dynamics of virus dispersion in the city of São Paulo, Brazil, following the reintroduction of an infected agent on the 138th day, using the official timeline (Table 1) of social distancing.

In Scenario 2, the social distancing rate after the reintroduction was set at 20%, and the simulation yields in Figure 4 show that the second wave is higher than the first. This could lead to an increased risk of collapse in the health system. In this scenario, at the end of 238 simulated days, we would have 19.67% of the population infected by the coronavirus, and 0.18% dying from the virus (0.92% lethality).

**Figure 5:**
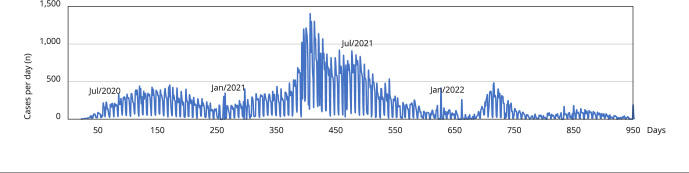
Daily cases of infection in the city of São Paulo, Brazil.

Source: São Paulo Data System Analysis ^3^.

Scenario 2 matches the dynamics of the spread of COVID-19 in the city of São Paulo ^3^, as shown in Figure 5, with two peaks in the infection curve, with the second peak being higher than the first. However, the dates of this phenomenon are not synchronized, which is predictable, since many variables interfere in the development of the pandemic.

**Figure 6:**
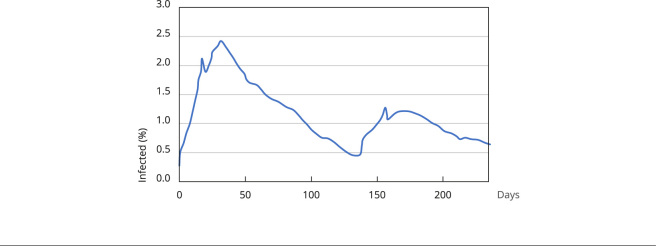
Dynamics of virus dispersion in the city of São Paulo, Brazil, after the reintroduction of an infected agent on the 138th day, using the official timeline (Table 1) of social distancing, extended by 100 days at 40% isolation rate.

In Scenario 3, the reintroduction of one infected person on the 138th day of the simulation was accompanied by a 40% social distancing rate for 100 days. Although we can see in Figure 6 that this also triggers a second wave of a COVID-19 outbreak, because social distancing remains at a higher level than in Scenario 2 (40% versus 20%), the second wave is almost always lower than the first one. At the end of 238 simulated days, we would have 15.07% of the population infected by the coronavirus, and 0.13% of the population dying (1.95% of the total infected).

In all scenarios, even with a lower rate of social distancing, the maintenance of other types of NPI can help keep the transmission rate low. This, in turn, can maintain a scenario of protection for longer and reinforce the exhaustion of contagion networks for the city of São Paulo.

As discussed above, many studies have shown the efficacy of social distancing, hand, washing, and using protective masks to slowdown the spread of the virus ^41^ ^,^ ^42^ ^,^ ^43^ ^,^ ^44^. In particular, the use of masks (which became mandatory in the state of São Paulo as of May 7, 2020) has been shown to reduce the intensity of COVID-19 itself.

## Discussion and conclusions

This work presents preliminary studies of the dynamics of the coronavirus epidemic curve for the city of São Paulo in the first year of the pandemic outbreak, using a simple and instrumental model. Our deliberate choice of working with limited parameter values for São Paulo shows the possibility of replicating the observed behavior of the epidemic without resorting to ad hoc hypotheses, such as herd immunity, in a population with a low infection rate.

Although models based on a deterministic SIR model can provide precise and comprehensive insights into various factors that influence the virus spread ^45^ ^,^ ^46^, this type of model (mean-field-like compartmental models) considers that an epidemic process evolves only when the density of susceptible individuals surpasses a certain threshold value. Moreover, these models are dependent on ad hoc parameters (such as the transmission rate) that are not grounded in empirical evidence. Although such models can offer high accuracy for a specific set of data, they can pose difficulties in comprehending and manipulating them without prior knowledge of the relevant scientific domain, unlike multi-agent models ^47^.

The MD Corona accurately predicted (July 12, 2020) the reduction in the number of cases and deaths in the municipality of São Paulo, in concurrence with the official data from the São Paulo State that indicated a slight decrease in the incidence of infections, deaths, and hospital bed occupancy ^3^. In addition, as illustrated in Figure 4, the outbreak of a new wave was higher than the previous one ^3^.

The more complex deterministic models ^48^ ^,^ ^49^ ^,^ ^50^ ^,^ ^51^ ^,^ ^52^ simulated the decrease in cases in July 2020; however, they did not describe the new waves and instead predicted a premature end to the pandemic. Nevertheless, another study ^14^ also correctly predicted this phenomenon.

The decrease in the number of infected agents is a counterintuitive outcome, given that the opening of the economy, in conjunction with a slight decrease in the social distancing index, would lead to an increase in the epidemic curve, since the municipality is far from the city to achieve the so-called group herd immunity.

The model reveals the existence of “local protection bubbles” against coronavirus infections, which means that susceptible individuals are shielded by a local barrier of immune individuals. This phenomenon may be further explained by the notion of exhaustion of the contagion network, despite an initial surge in the pandemic outbreak. This may be attributed to the prevalence of social distancing measures, as well as widespread preventive practices in society, which slow down the transmission rate. In other words, this exhaustion is due to the formation of protective bubbles and certain routines within the network of individuals maintaining social contact among themselves.

Given the low number of immune people in the system, this reduction represents an unsteady equilibrium that differs fundamentally from the anticipated stability of herd immunity.

These protective bubbles are susceptible to bursting, and subsequent waves of transmission may occur if social distancing measures are not adequately maintained, or if the virus is reintroduced into regions where a limited number of individuals have been infected. Accurately predicting the occurrence of such “burst bubbles” is extremely difficult without a policy of testing the population in the different districts of the municipality.

There are other possibilities that can be explored within this model, such as modifying the average immunity time for the population, increasing or decreasing the average effective transmission probability of individuals, and varying the number of people initially infected (or reintroducing more infected agents during the simulation).

Finally, it is noteworthy that the simplicity and accessibility of our model enable us to effectively demonstrate the impact of varying vulnerabilities in different territories on the epidemic curve ^53^. As evidenced by our findings, once the model is calibrated with region-specific data, we are able to generate reasonable predictions ^34^ ^,^ ^54^ ^,^ ^55^.
